# Supplementary material for: Effect of TSH stimulation protocols on adequacy of low-iodine diet for radioiodine administration
Source: PLoS One. 2021 Sep 7;16(9):e0256727. doi: 10.1371/journal.pone.0256727 (PMC8423307; doi:10.1371/journal.pone.0256727)
Supplement: Attachment — Submitted filename: Response to Reviewers.docx [file pone.0256727.s002.docx]

**Response to Reviewers:**

First of all, we appreciate all the reviewers’ helpful comments. We have made changes to the manuscript according to the reviewers’ comments. Below, please find our point-by-point responses to the comments. The reviewers’ comments are in *blue italics*, and our responses are in regular text, with changes to the manuscript in red. We hope that our revised manuscript is suitable for publication in the *PLoS One*.

(We changed the order of Figs. 1 and 2 according to the changes in main text.)

**Reviewer #1**

*1. Just minor correction of thyroid globulin in the introduction (to replace it by thyroglobulin TG)*

We appreciate the reviewer’s comment and corrected the error.

**Reviewer #2**

**Major**

[Statistical Analysis]

*1. If the data are normally distributed as implied by the use of means and standard deviations, non-parametric tests should not be used and a re-analysis using t-tests and ANOVAs is necessary.*

As the reviewer commented, ‘UICR level’ was a continuous variable and showed normal distributions. In our previous manuscript, statistical methods used for comparing UICR levels were missing, and we added them. Non-parametric tests were used for ordinal variables.

(Page 5, line 121-126) In comparison of UICR values, *t*-test was used for two-group comparisons (THW vs. rhTSH; male vs. female; ILD-experienced vs. ILD-naïve) and ANOVA was used for multiple-group comparison (in terms of clinical factors). In comparison of ordinal variables like UICR adequacy, Mann-Whitney *U* test and Kruskal-Wallis H test were used for two-group and multiple-group comparisons, respectively.

*2. There needs to be more specific details given about the comparisons being made (eg. line 100 – what two groups; line 103 what repeated measurements?)*

We revised the manuscript to describe more specifically the comparisons that we performed. Please refer to our response to the above comment 1.

*3. I am finding it difficult to understand the explanation of the multivariate analysis. Exactly which variables were entered and why?*

We attempted to determine which factor is independently significant to achieve ‘excellent’ adequacy class of LID. We included such variables that are significant for determining ‘excellent’ adequacy of LID in univariate analyses. Finally, sex (male vs. female), age (< 50 vs. ≥ 50 years), prior LID experience (yes vs. no), procedure (^123^I scan vs. ^131^I treatment) and TSH stimulation protocol (THW vs. rhTSH) were included in the multivariate analysis. We clarified the method and included variables in the manuscript.

(Page 5, line 127-129) Multivariate analysis using stepwise regression was performed to determine independent and significant factors for achieving ‘excellent’ adequacy of LID, with including variables that were significant in univariate analyses.

(Page 10, line 200-203) Multivariate analysis was performed to determine significant determinants for achieving ‘excellent’ adequacy class of LID, including variables of sex (male *vs*. female), age (< 50 *vs*. ≥ 50 years), prior LID experience (yes *vs*. no), procedure (^123^I scan *vs*. ^131^I treatment) and TSH stimulation protocol (THW *vs*. rhTSH).

[Results]

*4. A comparison of urinary iodine in patients prepared with rhTSH vs THW was, I think, the primary research question (and seems to be the most important factor in whether or not someone achieved excellent depletion). Therefore, it seems odd to present the urine iodine levels according to demographic and clinical factors first and completely independently of preparation. I suggest “urine iodine levels according to TSH stimulation protocol” be presented immediately after the demographics. The comparison of demographic and clinical factors can then be presented.*

We changed the order of paragraphs according to the reviewer’s recommendation.

(Page 7–10)

*5. Information should be given on the number of patients in each clinical group who were prepared with rhTSH vs THW and the corresponding mean/median urinary iodine measures (add this to table 2).*

We added the data, according to the reviewer’s comment.

(Table 4) UICR according to demographic and clinical factors

[Discussion and conclusion]

*6. The authors should be much more cautious in recommending that short-term THW be considered alongside rhTSH because this study has not demonstrated that the proposed protocol would effectively lower urinary iodine more than rhTSH alone, or that there would be any impact on success rates. It could be a recommendation for future research. Some evidence is presented from Barbero et al, a study, that did analyse treatment outcomes between various preparatory protocols (although the study used furosemide, which is not usually recommended) to support the recommendation. However, the authors should also consider and discuss evidence from Tala Jury et al (citation 12) that reported “…no significant difference in UIE between ablated or nonablated patients both in the whole group and the rhTSH or THW groups” and from the HiLo and ESTIMABLE1 trials, large RCTs that found no evidence for a difference in outcomes at 6-8 months and 5-year follow-up, between patients prepared with rhTSH vs THW. Line 238. Rather than recommending an intervention that they haven’t tested which is more disruptive for patients, could the authors comment on whether improved dietary advice for those receiving rhTSH would achieve the desired results?*

We agree with the reviewer. Although the UICR level and achieved LID adequacy were better with THW than with rhTSH, additional evidence is required regarding the effect of short-term THW on UICR level or RAI treatment outcome. According to the reviewer’s comment, we revised the manuscript as shown below. However, additional diet modification is difficult in patients with rhTSH use, because instruction for iodine restriction diet is already considerably strict in our institution and many hospitals.

(Page 13, line 260-268) The main finding of the present study is in line with the previous results that continued thyroid hormone replacement with rhTSH use can affect body iodine. Although most of patients were within ‘excellent’ or ‘adequate’ range of UICR with rhTSH, there was a significant difference in UICR values between THW and rhTSH groups. This difference may affect efficacy of RAI treatment particularly in those who need maximal efficacy of RAI treatment (such as high-risk patients) or those who are prone to poor LID adequacy (such as old patients). Thus, further researches are warranted to find out effective methods to reduce body iodine pool in case of rhTSH use. Additionally, the efficacy of such methods on outcomes of RAI treatment needs to be evaluated.

*7. Given that evidence that very low iodine status enhances treatment success is very weak, I would like to see an explanation of why no analysis was made of the outcome of ablation or treatment during this study. As this is a retrospective study the data should be available.*

We agree with the reviewer that the treatment efficacy should be the final endpoint. However, our study was initially focused on the effect of TSH stimulation protocols on UICR, and designed to include somewhat heterogeneous situations including I-123 scan, RAI ablation and treatment of various doses. Additionally, repeated scans or RAI treatments were all included. Thus, relevant analyses on the ablation success rate or RAI treatment efficacy were not evaluated in our study. As the reviewer commented, we mentioned that the treatment efficacy should be investigated in future researches, and we also mentioned it as a limitation of our study.

(Page 13, line 266-268) Thus, further researches are warranted to find out effective methods to reduce body iodine pool in case of rhTSH use. Additionally, the efficacy of such methods on outcomes of RAI treatment needs to be evaluated.

(Page 14, line 296-299) Finally, differences in outcomes of ablation or treatment were not analyzed in this study. Because the main purpose of this study was to compare LID efficacy between two different TSH stimulation protocols, all repeated scans and RAI treatments were included in this study.

*8. The abstract would need re-writing to reflect these comments.*

We rewrote the abstract.

**Minor**

[Introduction]

*1. Define intermediate and high-risk thyroid cancer and high-dose ^131^I.*

Because the definition criteria of each risk group is a somewhat long list, please refer to the reference paper of ATA guidelines (Ref. 1). We added a usual definition for high-dose ^131^I (≥ 3.7 GBq) as shown below. However, in our study we classified RAI treatments with a cutoff of 1.2 GBq.

(Page 2, line 57) high-dose ^131^I treatment (usually, ≥ 3.7 GBq)

*2.* *Line 47-49 (Additionally, the thyroid globulin level when thyroid stimulating hormone (TSH) is stimulated is the most sensitive biomarker for differentiated thyroid cancer.) Remove, this observation is not relevant to this paper.*

We revised the manuscript according to the reviewer’s comment.

*3. It would be helpful to set out the research question(s) much more clearly and to provide some explanation as to why the analyses of other clinical factors were undertaken.*

We revised the manuscript according to the reviewer’s comment.

(Page 2-3, line 74-76) The aim of the present study is to evaluate the influence of the different TSH stimulation protocols on the adequacy of LID. The influence of various clinical factors on the adequacy of LID were also investigated.

[Methods]

*4. Please provide a full description of the inclusion and exclusion criteria.*

We rephrased the manuscript to clearly describe the inclusion and exclusion criteria.

(Page 3, line 83-86) The inclusion criteria were as follows; patients with thyroid cancer who underwent LID for ^131^I treatment or ^123^I scan between 2016 and 2020, and available UICR measurements conducted for evaluating the adequacy of LID.

(Page 3, line 96-97) The exclusion criteria were as follows; UICR measurements of children less than 10-year-old at the time of measurement, and outlier results > 1000 μg/g Cr.

*5. There are substantial variations in the LIDs advised between different countries and institutions. It would be useful to include a table showing the advised LID used in this study.*

According to the reviewer’s comment, we added a new table to show LID instruction of our institution.

(Table 1) Instructions of LID for RAI scan or treatment

*6. Statistical significance is a function of sample size – it is better to give the precise p-values (which has been done here), to talk about levels of evidence instead of referring to a result as ‘significant’ and to consider the effect size (see https://www.ncbi.nlm.nih.gov/pmc/articles/PMC1119478/)*

In this manuscript, we decided to report P-values to 3 decimal places. Thus, the limit of reported P-value was 0.001. Almost all P-values were < 0.001 in the result, and one P-value that was larger than 0.001 was 0.013 and we reported the precise P-value for it.

[Results]

*7. Lines 1136 - 116: Cut text from 985 to 2 times. All of this is presented in table 1.*

According to the reviewer’s comment, we revised the manuscript to remove redundant description. The numbers of patients who underwent LID once or ≥ 2 times during the study period is different from the numbers shown in the Table.

(Page 6, line 140-142) 1045 (99.1%) were differentiated thyroid cancer, and 9 (0.9%) were poorly differentiated thyroid cancer. During the study period, 502 (47.6%) patients underwent LID once and 552 (52.4%) patients underwent LID more than once.

*8. Line 129, 132, 133, 136. Quote the effect not the p-values in the text. For example, line 129 “There was strong evidence that UICR was higher in women than in men (55.0 (52.8) vs 40.4 (47.1) ug/gCr)” is much more meaningful than just quoting the p-value.*

The values are shown in Table 2 in the previous manuscript. However, we revised the Table (now Table 4) and added the values in the text.

(Page 8, line 181-182; Page 8-9, line 184-186, 188-189) UICR was significantly higher in women than in men (55.0 ± 52.8 vs. 40.4 ± 47.1, P < 0.001, Table 4). … UICR was increased with age and there were significant differences between the age groups (35.0 ± 31.1, 45.9 ± 47.2, 56.1 ± 50.3, and 83.3 ± 86.6 for each age group, respectively, P < 0.001, Table 4). … UICR was significantly higher in the first LID than that in second or more LID (55.3 ± 44.2 vs. 46.6 ± 56.6, P < 0.001).

*9. Retitle table 2 and the corresponding heading ‘according to demographic and clinical factors’.*

We revised the title of the table (now, Table 4).

(Table 4) UICR according to demographic and clinical factors

*10. Consider removing the figures and putting the information into table 2.*

We understand the reviewer’s concern. However, we believe the figures are helpful for readers to easily see the results, and we want to show the figures.

*11. The title of table 3 doesn’t match what the table is showing. Also, it needs to be clearer that the numbers in each cell in table 3 refers to numbers of patients.*

We revised the title of Table 3, and also its format.

(Table 3) The number of patients according to TSH stimulation protocols and LID procedures in those who underwent the first and second LID during the study period

[Discussion]

*12. Please start the discussion with the main findings, not information about whether urine iodine creatinine is a good measure (that can go under limitations).*

We revised the manuscript according to the reviewer’s comment.

(Page 11, line 215-223) In the present study, UICR was analyzed in a large cohort including 1715 measurements of 1054 patients, and it was demonstrated that UICR is significantly associated with TSH stimulation protocol. In most cases (92.7%), 2-week LID resulted in ‘excellent’ or ‘adequate’ range of UICR. However, UICR was higher and proportion of ‘excellent’ adequacy was lower in patients who used rhTSH without THW compared to those used THW. In patients who underwent repeated LID using both rhTSH and THW protocols, UICR was higher in case of rhTSH, regardless of the order of used protocols. Furthermore, TSH stimulation protocol was a significant and independent determinant for achieving ‘excellent’ adequacy of LID.

*13. “….body retention of RAI increases with use of LID”. This effect has been observed elsewhere (Maruca et al., 1984) and is not desirable (although Plujimen et al do not consider it to be highly problematic). Is this what the authors wanted to communicate from this paper?*

We appreciate the reviewer’s comment. We intended to mean increased retention of RAI in remnant thyroid or cancer tissue as reported by Pluijmen et al. We revised it as follows, so that the meaning is clear.

(Page 12, line 230-231) such as low urine iodine level and high 24-hour neck RAI uptake in LID [12].

*14. Please amend to show the study findings more completely ie that success rate was similar (about 80%) for all patients with UICR <250mg/gCr, not just those <50mcg/gCr. Sohn et al cannot be used to suggest that a UICR <50mcg/gCr is superior to <100mcg/gCr.*

We revised the manuscript according to the reviewer’s comment.

(Page 12, line 231-234) In a relatively recent study, ablation success rate was 50% in patients with UICR ≥ 250 μg/g Cr whereas it was 76–82% in patients with UICR < 50 μg/g Cr, although the ablation success rates of those with UICR < 250 μg/g Cr and those with < 50 μg/g Cr were similar [13].

*15. Line 211 The European Thyroid Cancer Taskforce made that recommendation in 2006 and it does not reflect guidelines from other European bodies which mainly advise 1-2 weeks (eg European Association of Nuclear Medicine in 2008 recommended 1-2 weeks (Luster et al) – please add this reference. (And Pluijmen et al, cited as a study that demonstrated LIDs improve ablation only used 4 days – perhaps comment on that).*

We revised the manuscript according to the reviewer’s comment. The 4-day protocol used by Pluijmen et al. has been cited in Line 231.

(Page 12, line 242-244) While the American Thyroid Association and the European Association of Nuclear Medicine recommend 1 to 2-week LID prior to RAI administration,

*16. Line 241 – 252. There is some discussion of the demographic and clinical differences, can the authors comment on whether changes to how dietary advice is delivered locally could help with these differences.*

It may be considered to give intense education or instruction to old-aged patients or those who are scheduled for ^131^I treatment. However, because the main finding of this study is to compare those with rhTSH use and THW, we suppose it would be better not to include this comment in the manuscript.

*17. I have not extensively proof-read the article or corrected minor errors in English but there are some and I suggest a proof reader be employed*

We appreciate the reviewer’s comment. We have requested additional proof-reading and corrected several points of errors.
